# Early retention among pregnant women on ‘Option B + ’ in urban and rural Zimbabwe

**DOI:** 10.1186/s12981-021-00333-3

**Published:** 2021-04-01

**Authors:** Anesu N. Chimwaza, Hannock Tweya, Owen Mugurungi, Angela Mushavi, Solomon Mukungunugwa, Ngwarai Sithole, Justice Nyakura, Mbazi Senkoro, Philip Owiti, Ronald Ncube, Talent Tapera, Winnie Mandewo, Jeffrey K. Edwards, Aveneni Mangombe, Isaac Taramusi

**Affiliations:** 1 National AIDS and Tuberculosis Unit, Ministry of Health and Child Care, Harare, Zimbabwe; 2 National Reproductive Health Unit, Ministry of Health and Child Care, Harare, Zimbabwe; 3 Department of Global Health, University of Washington, Seattle, Washington USA; 4 International Union Against Tuberculosis and Lung Disease, Harare, Zimbabwe; 5 Lighthouse Trust, Lilongwe, Malawi; 6 National Tuberculosis, Leprosy and Lung Disease Program, Nairobi, Kenya; 7 International Union Against Tuberculosis and Lung Disease, International, Paris, France; 8 Institution: National Institute for Medical Research – Muhimbili Centre, Dar es Salaam, Tanzania; 9 National AIDS Council, Harare, Zimbabwe; 10 Elizabeth Glaser Pediatric AIDS Foundation, Harare, Zimbabwe; 11 AFRICAID Zvandiri Harare, Harare, Zimbabwe

## Abstract

**Background:**

In 2013, the World Health Organisation (WHO) recommended Option B+ as a strategy to prevent mother-to-child transmission (PMTCT) of HIV. In option B+ , lifelong antiretroviral therapy (ART) is offered to all HIV positive pregnant and breastfeeding women to reduce MTCT rate to less than or equal to 5%. Its success depends on retaining women on ART during pregnancy, delivery and breast-feeding period. There is limited data on early retention on ART among pregnant women in Zimbabwe. We therefore assessed early retention among women on Option B + from antenatal care (ANC) until 6 months post ANC booking and at delivery in Bulawayo city and Mazowe rural district of Zimbabwe.

**Methods:**

We collected data for pregnant women booking for ANC between January and March 2018, comparing early retention among ART naïve women and those already on ART. The two cohorts were followed up for 6 months post ANC booking, and this was done in two districts. Data were collected from routine tools used at facility level which include ANC, delivery and ART registers. The Kaplan-Meier survival analysis was used to estimate retention probabilities at 1, 3 and 6 months post-delivery and for retention at delivery proportions were used. Poisson regression was used to investigate factors associated with non-retention at 6 months post ANC booking.

**Results:**

A total of 388 women were included in the study with median age of 29 years (IQR: 25–34). Two-thirds booked in their second trimester. Retention at 3 and 6 months post ANC booking was 84% (95% CI 80–88) and 73% (95% CI 69–78) respectively. At delivery 81% (95% CI 76–84) were retained in care, 18% lost-to-follow-up and 1% transferred out. In this study we did not find marital status, gestation age, facility location, ART status at ANC booking, to be associated with loss to follow-up.

**Conclusion:**

In this study, we found low retention at 3, 6 months and delivery, a threat to elimination of Mother-to-child Transmission of HIV in Zimbabwe. Our findings emphasize the need for enhanced interventions to improve early retention such as post-test counselling, patient tracing and visit reminders.

## Plain English summary

In 2013, the World Health Organisation (WHO) recommended Option B + as a policy to prevent mother-to-child transmission (PMTCT) of HIV, by offering lifelong antiretroviral therapy (ART) to all pregnant and breastfeeding women. For this policy to succeed it depends on retaining women during pregnancy and breastfeeding period. Zimbabwe has been implementing this policy since 2013 but there is limited data on early retention on ART among pregnant women. In this study we assessed early retention among pregnant women on ART from antenatal care (ANC) until 6 months post ANC booking and at delivery in Bulawayo city and Mazowe rural district.

Data were collected from facility health registers and patient ART booklets. The study results demonstrated that there is low retention on ART among pregnant women post-ANC booking, decreasing from 89% by the first month to 73% by month six. At delivery 19% of the women were lost to care, i.e. no longer coming to the facility for resupply of HIV medicines.

It is evident from these results, that the goal of eliminating HIV in children by 2022 in Zimbabwe is unlikely to be achieved. Therefore, the PMTCT program should consider interventions to improve early retention such as enhanced post-test counselling, patient tracking and visit reminders.

## Background

Approximately 1.3 million women living with HIV (WLHIV) become pregnant every year globally and have a 15–30% risk of transmitting HIV to their infants, during pregnancy, delivery or breastfeeding [1, 2] Antiretroviral therapy (ART) and other interventions can reduce the risk of transmission to less than 5% in breast feeding countries and less than 2% in non-breast feeding countries [2].In 2013, the World Health Organisation (WHO) recommended Option B + as a strategy to prevent mother-to-child transmission (PMTCT) of HIV [1]. In Option B + , lifelong ART is offered to all pregnant and breastfeeding women, regardless of their CD4 count level or WHO clinical stage and 6 weeks of daily Nevirapine for the infant [1].

The success of Option B + depends on retaining women throughout the continuum of care during pregnancy and breastfeeding period [3]. Early retention in the first 6 months is an important measure of PMTCT programme success and overall quality [4]. A retrospective study in Malawi noted that early retention after 1 and 3 months decreased from 86 to 73% respectively [5]. Among pregnant women enrolled on Option B + in an Ethiopian cohort, high early attrition was a major problem, with only 88% being retained at 6 months [6]. In Ghana, 66% of pregnant women enrolled into PMTCT from ANC booking, at delivery, and at least one exposed infant follow-up visit completed the cascade [7]. Retention in care generally decreases with time from ART initiation as shown by a systematic review of studies done in Africa [8]. The retention decreased from 90% in the first month to 80% by month three [8]. WHO encourages countries implementing Option B + to constantly measure the early retention rates at 1 and 3 months for all pregnant WLHIV [4].

Zimbabwe is one of the 22 high burden countries which account for over 90% of all pregnant women with HIV worldwide [2]. Implementation of Option B + started in 2013, however, in 2017, the country launched an ambitious 5-year plan with a goal of eliminating mother-to-child-transmissions of HIV and syphilis by 2022 [2, 9]. ART coverage among pregnant WLHIV increased from 84% in 2013 to 95%in 2017, including WLHIV newly enrolled on ART (ART naïve) and those already on ART at ANC booking [10]. Although previous studies in Zimbabwe reported retention rates, the studies focused on 6-month and 1-year retention rates for ART naïve women at ANC booking [11, 12]. There is limited data on early retention in care (1, 3, 6 months) for ART naïve women and those already on ART post ANC booking.

The United Nations Programme on HIV and AIDS (UNAIDS) developed Spectrum model software for HIV, to assist countries to determine state of the epidemic, identify targets for programmes and measure impact of response [13]. The software relies on availability of valid HIV surveillance, survey and program data to generate key HIV indicators such as, the population in need of PMTCT, Mother-to-child Transmission (MTCT) rate and short‐term projections [13]. Zimbabwe has been using this model since 2009 with default ART retention rate values at delivery, estimated based on a review of literature to be 80% and 75% for WLHIV newly enrolled on ART and those already on ART before pregnancy respectively [14, 15]. Understanding early retention in care is essential for Zimbabwe to be able to reach the goal of having an MTCT rate of less 5% by 2022.

In this study, we describe baseline characteristics of WLHIV receiving ART and their 1, 3, 6 months retention in care post ANC booking and at delivery at maternal child health (MCH) clinics in Bulawayo city and Mazowe rural district of Zimbabwe.

## Methods

### Study design

This was a cohort study using secondary data from ART registers and patient records.

#### Setting

##### General setting

Zimbabwe is a landlocked country with a population of 13.6 million people and the majority (67%) lives in rural areas. The country consists of 10 provinces, 63 districts and 1, 722 public health facilities. Treatment for HIV and TB are free of charge with 1560 sites providing ANC and PMTCT services [16].Almost 93% of pregnant women aged 15–49 years accessed antenatal care (ANC) at least once and 72% of deliveries were done by skilled health workers [16].

##### National PMTCT programme in Zimbabwe:

The national PMTCT programme started in 1999 with 3 pilot sites and scaled up to 1560 health facilities by 2017 [17]. With the implementation of Option B + , pregnant women are encouraged to register for ANC as soon as they know that they are pregnant. ANC registration involves recording of baseline demographic data and screening for HIV and syphilis and doing all other assessments as guided by the national ANC protocol. HIV testing services are offered to all women through an “opt-out” strategy and those who decline testing at first ANC visit are offered the test at subsequent visits [18]. Information about HIV test results and ART status are recorded in the ANC, ART registers and opportunistic infection (OI)/ART patient care booklet [17]. HIV positive women are initiated on tenofovir, lamivudine and efavirenz (TDF + 3TC + EFV) as the preferred first-line regimen. ART naïve pregnant women are seen after 2 weeks of ART initiation in the first month, and then monthly for 4–6 months, and thereafter 3 monthly supplies for those with documented adherence [17]. Women who are already on ART at first ANC visit continue with their treatment and both ANC and ART visits are synchronised to optimize ART adherence. After delivery, the mother-infant pairs will continue to be followed-up at maternal child health clinics until 24 months or the cessation of breastfeeding. ART program outcomes such as alive and in care, opt out, transfer-out are updated during clinic visit while default and death are updated later.

#### Study sites and population

Data were collected from 18 facilities in Bulawayo city, the second largest city in Zimbabwe and 31 facilities in Mazowe rural district. All pregnant women living with HIV already on ART and newly initiating ART at first ANC visit at the study facilities, were eligible for inclusion in the study. Women who were initiated on ART at other facility and did not transfer to the study facility were excluded in this study, because they had no ART record.

#### Data variables and collection

Data were collected from the ANC register, O/I ART patient booklet and delivery register which were completed by trained health care workers. This included: the demographic details, ANC booking date, expected date of delivery, ART status, marital status, disclosure status, date of ART initiation, delivery date, program outcome and program outcome date. Validation was done using other facility registers such as the ART, exposed infant registers to get the delivery date and the Electronic patient monitoring system (EPMs). Pregnant women testing HIV positive or transferred in during their subsequent visits were excluded for this study. Data were first extracted from the ANC register to select all women that had booked for first ANC already on ART or those who tested HIV positive at first visit, The OI ART number was also extracted from the ANC register which was then used to pull out the OI ART patient booklet, which comprises all ART visits done by the patient from ART initiation. Data extraction proformas were used for data abstraction between December 2018 and March 2019. Data were entered in in Epi-Data version 4.1.1.0 and analysed in Stata.

For the purposes of this study, retention in care was defined as having attended an appointment as scheduled and picking up ARVs as prescribed. For ART naïve pregnant women their date of ANC booking was the same with the ART start date and slightly differed in some cases. For those already on retention was calculated from the ANC start date going to the date of last review. Lost-to-follow-up was defined as someone not having patient record or recorded patient visit in the patient OI ART patient booklet. Transfer out was defined as a patient formally transferred to another facility and opt out was defined for someone who refused to be initiated on ART [17]. Retention at delivery was defined as a patient still in ART care from the day they were booked for ANC until they deliver, and the sensor date used was the delivery date.

Patients without a delivery date or their last ART visit was before they delivered, were considered to be lost-to- follow-up, since there was no record of them showing that there are still in care.

### Data analysis

Descriptive statistics were performed including median and inter quartile range (IQR) for the women’s age, and frequencies for categorical variable to explore baseline characteristics of the study population. In time-to-event analysis, observation time began at first ANC visit and ended at either default, transfer to another ART facility or at 6 months post first ANC visit, whichever occurred first. The Kaplan- Meier methods were used to estimate proportion of women retained at 1, 3- and 6-months post first ANC visit and the sensor date used was the last visit date. Proportions were used to calculate the retention at delivery and the sensor date used was the delivery date. Poisson regression models were used to investigate factors associated with non-retention at 6 months post first ANC visit. A significance level of p < 0.05 was used throughout.

## Results

### Demographic characteristics of study participants

**Fig. 1:**
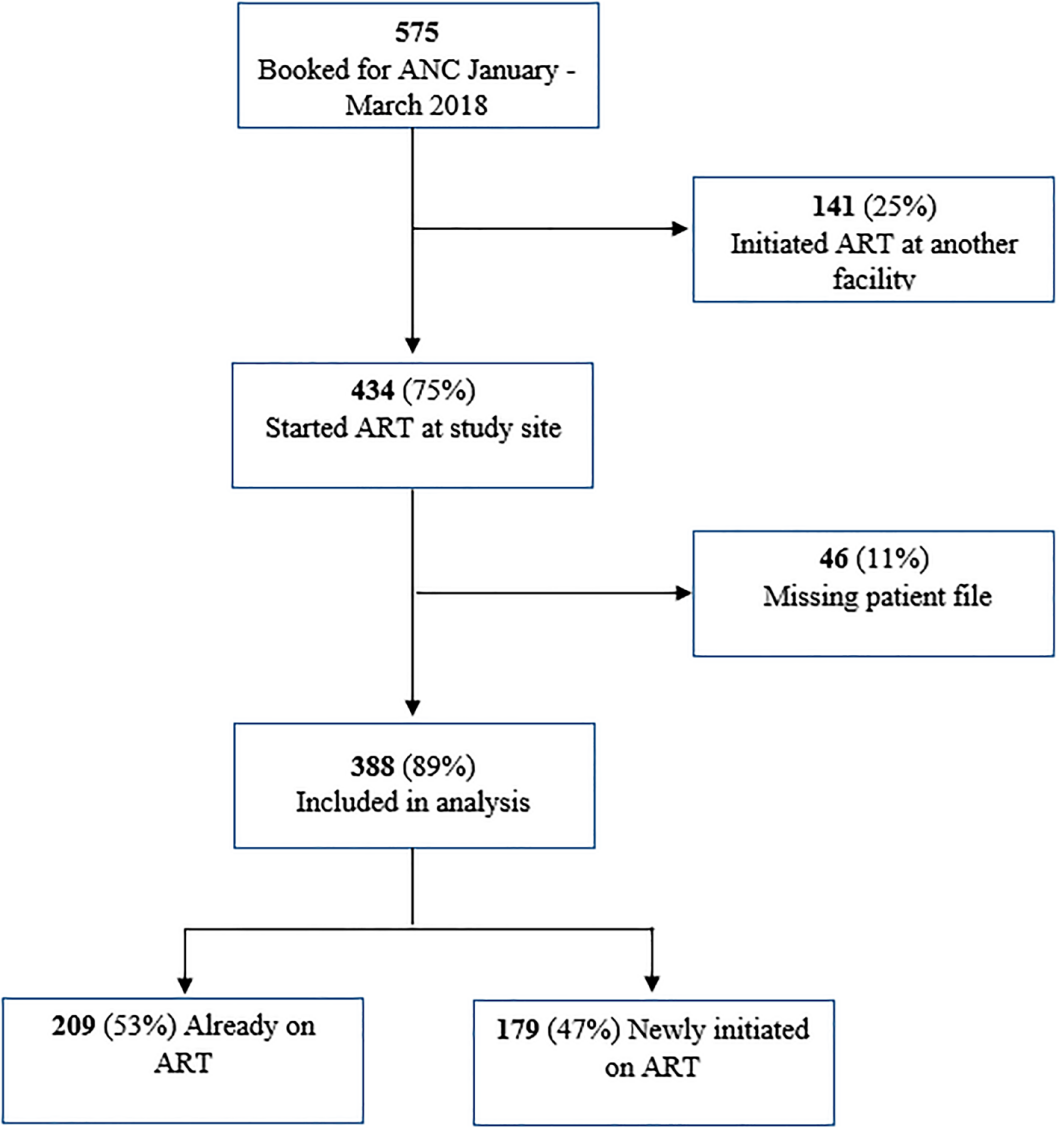
Study participants recruited from pregnant women living with HIV booking for ANC: Zimbabwe

Between January and March 2018, 575 WLHIV booked for ANC (Fig. 1). Of these, 187 (33%) women were excluded from the study because their files were missing: 141 (75%) initiated ART at other facilities prior to ANC booking at the study facilities and 46 (25%) initiated ART at the study facility. A total of 388 women were included in the study analysis and the characteristics of women included in the study were not statistically different from those who were excluded. Among those enrolled, 209 (54%) booked for ANC while on ART. Table 1 summarizes characteristics of participants enrolled in the study. The median age was 29 years (IQR: 25–34) with two-thirds booked for ANC during their second trimester. Seventy percent of the women reported to be married and 72% booked for ANC at urban facilities.

**Table 1:** Characteristics of HIV positive pregnant women booking for ANC in Bulawayo city and Mazowe district

Characteristic	N	%
Eligible for the study	388	
Age		
15–19	21	5.4
20–24	79	20.4
25–29	107	27.6
30–49	181	46.6
Parity		
0	64	16.5
1	97	25
2	111	28.6
3+	115	29.6
Missing	1	0.3
GA at first ANC visit (weeks)		
< 12	45	11.6
13–27	237	61.1
28+	76	19.6
Missing	30	7.7
Marital status		
Single	35	9
Married/living together	272	70.1
Widowed/divorced/separated	18	4.7
Missing	63	16.2
HIV status disclosure to someone		
Yes	148	38.1
No	18	4.7
Missing	222	57.2
Facility location		
Mazowe District (Rural)	278	71.6
Bulawayo City (Urban)	110	28.4

*HIV* Human immunodeficiency virus, *GA* gestational age, *ART* antiretroviral therapy, *ANC* antenatal care

### ART retention post ANC booking

**Fig. 2:**
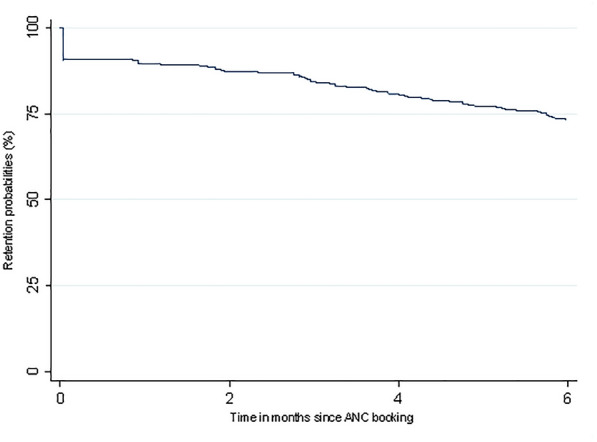
The Kaplan–Meier estimates for retention among women living with HIV booking for first ANC in Zimbabwe

Retention on ART at 1 month post ANC booking was 89%, 84% by month 3, declining to 73% by the sixth month (Table 2). Women who were newly initiated on ART during pregnancy had generally higher retention in the first three months post ANC booking compared to women who were already on ART at booking., though the differences were not statistically significant. As reflected in the Kaplan- Meier curve (Fig. 2), some women who were lost to follow-up had no follow-up visit and retention decreased steadily within six months post ANC booking. At 6 months post ANC booking, 284 (73%) of the women were retained in care, 101 (26%) defaulted treatment and 3 (1%) had transferred to another facility. Overall rate of lost to follow-up was 5.3 per 100 person-month (95% CI 4.4–6.5). At delivery, 101 (18%) had defaulted treatment and 3 (1%) transferred out. The overall retention at delivery was 81% (95% CI 76–84). Retention at delivery for pregnant women already on ART was 78% (95% CI 72–83) compared to 84% (95% CI 78–89) for ART naïve pregnant women.

**Table 2:** Outcome at 1, 2,3 and 6 months for HIV positive pregnant women receiving antiretroviral therapy

Months After first ANC booking	Overall retention			Already on ART			Newly initiated on ART		
	N	Rates/100 person-month	[95% C.I]	N	Rates/100 person-month	[95% C.I]	N	Rates/100 person-month	[95% C.I]
Total	388			209			179		
Month 1	348	89	(0.86–0.92)	182	87	(0.81–0.91)	167	93	(0.88–0.96)
Month 2	340	87	(0.84–0.90)	179	85	(0.80–0.89)	162	90	(0.85–0.94)
Month 3	330	84	(0.80–0.88)	177	83	(0.78–0.88)	153	85	(0.79–0.89)
Month 6	284	73	(0.69–0.78)	154	73	(0.67–0.79)	134	74	(0.67–0.79)

*ART* antiretroviral therapy, *ANC* antenatal care, *CI* confidence interval, *HIV* human immunodeficiency virus

Table 3 shows assessment of associations between characteristics of option B + women and ART default at 6-month post ANC booking, notably, none was associated with default.

**Table 3:** Factors associated with discontinuing ART at delivery HIV positive pregnant women on antiretroviral therapy in Bulawayo city and Mazowe district: Zimbabwe Jan-Mar 2018

Characteristics	Number on ART	Number (%) Attrition		Unadjusted RR (95% CI)	P value	Adjusted RR (95% CI)	P value
Age (years)							
15–19	16	5	7%	Reference		Reference	
20–24	66	13	17%	0.83 (0.3–2.1)	0.72	0.64 (0.2–2.1)	−0.46
25–29	83	24	32%	1.1 (0.4–3.0)	0.8	0.8 (0.3–2.8)	0.79
30–49	148	33	44%	0.82 (0.3–2.1)	0.68	0.6 (0.2–2.1)	0.44
Parity category							
0	52	12	16%	Reference		Reference	
1	78	19	25%	1.0 (0.5–2.1)	0.91	1.0 (0.4–2.6)	0.97
2	91	20	27%	0.9 (0.4–1.8)	0.82	0.9 (0.3–2.6)	0.89
3+	91	24	32%	1.9 (0.5–2.2)	0.79	1.0 (0.3–2.8)	0.93
Gestational age at first ANC visit (weeks)							
≤ 12	31	14	19%	Reference		Reference	
13–27	196	41	55%	0.7 (0.3–1.2)	0.26	0.6 (0.3–1.2)	0.16
≥ 28	65	11	15%	1.3 (0.6–2.7)	0.58	0.9 (0.3–2.3)	0.78
Marital status							
Married/living together	222	50	67%	Reference		Reference	
Single	29	6	8%	1.0 (0.4–2.4)	0.91	1.1 (0.41–2.8)	0.90
Widowed /Divorced/Separated	13	5	4%	2.2 (0.7–7.2)	0.17	1.4 (0.52–3.8)	0.51
Facility Location							
Urban	180	44	59%	0.7 (0.5–1.1)	0.16	0.7 (0.4–1.3)	0.27
Rural							
ART status							
Already on ART	150	29	39%	1.3 (0.8–2.1)	0.27	1.6 (0.8–2.9)	0.17
Newly initiated on ART							

## Discussion

This is the first study in Zimbabwe to assess early retention among women on ART at first ANC booking until six months post ANC booking. The retention rates in the first three months of follow-up decreased from 89 to 84% post ANC booking. Just over a tenth of WLHIV registering for first ANC visit never returned for follow-up after month one, the proportion gradually increasing to 27% by the sixth month. The results show that ART naïve pregnant women were more likely to continue treatment if they retain after month one than those already on ART.

Compared to other studies, six-month retention observed in this study was lower than those reported in Malawi [18] and Ethiopia [6] that reported 82% and 88%, respectively, demonstrating challenges in early ART retention in Zimbabwe. The three month retention observed in this study was similar to that reported in Malawi and Nigeria (84%) [18]. Retention at delivery for ART naïve and those already on ART are slightly higher than those found in the Rwanda (75%) and Haiti (80%) [15, 19]. The possible reason why the rates are slightly high is because of the different study settings, since the two were done under Option B settings and the study settings was a mature Option B + setting. We, therefore, suggest strategies for consideration to address the low ART retention.

First, our findings highlight the importance of paying attention to the initial period soon after ANC booking for the success of option B + . Approaches to improve early ART retention through facility and community engagement are needed. For the facility, the four steps outlined in the operational and service delivery manual should be adhered to, which include giving basic HIV and ART education, taking clinical history, plan to complete ART sessions, and agree on a treatment plan [17]. Moreover, more resources are needed to strengthen on-site routine support and mentorship programmes with special focus to enhancing ART retention. Since retention rapidly decreases within the first 6 month after ANC booking, repeated adherence counselling and reminding women on ART of their clinic appointments via phone calls, SMS text messaging should be considered, as they have been proven effective in increasing retention in several studies [8]. Community engagement is one of the strategies that has been proven to increase early retention in care [12]. Implementing these strategies such as formation of mentor mothers, lead mothers or fathers, male (champions) mobilisers and support groups as outlined in the enrolment and adherence (EAR) package for HIV program in Zimbabwe, has a potential to improve early retention of pregnant women [20]. The formation of these groups in all facilities could contribute meaningfully to the MTCT elimination agenda.

Second, the study showed that women who started ART at other facilities and registered for ANC at the study facility had no OI/ART patient booklet. It is unclear if these women received ARVs after ANC booking and on subsequent visits. The presence of accurate and complete information in patient booklets provides evidence of the services the patient receives during a clinic appointment. Moreover, it is an effective mode of communication across providers in the continuum of care. The PMTCT program should consider adding a column in the ANC register to track the ART follow-up status at each visit (active on treatment, default, lost to follow-up), this will assist in monitoring pregnant women already on ART but taking their ART at other facilities. There is need also to add the delivery date in the pregnancy column of the patient ART booklet. This could assist the health workers to document comprehensive information about those women in one source document and makes it easy to distinguish ART visits before and after delivery. The PMTCT program should develop a monitoring system to assess the availability and completeness of documents for all pregnant women initiated on ART treatment.There is an urgent need for the country to expand the use of the existing electronic systems such as (ePMS, EHR, PMTCT tracker) to all sites which will enable longitudinal follow-up of mother during the ART follow-up.

Finally, the study results also reflect that most pregnant women book for ANC in the second trimester, considered too late for an effective PMTCT programme [20]. There is urgent need for community engagement programmes to promote early booking. This can be done through radio programmes and different community sensitisation gatherings.

The major strength of this study was that it assessed retention within routine PMTCT programme setting therefore the results reflect real life operational challenges in MCH clinics in Zimbabwe. However, our study should be considered with the following limitations. First, we used routine data from the patient ART card and some information was missing. The effect of this may be minimal as the differences between women who were included and those excluded from the study were not statistically significant. Second, data were collected from selected clinics in only two districts which may not be representative of the population of PMTCT women in Zimbabwe. Finally, we could not verify true outcomes of patients who were classified as loss to follow-up. Some women might have been misclassified as loss to follow up, due to missing delivery dates, undocumented transfer out or deaths.. Despite these limitations, we believe that the study findings are useful to inform PMTCT program implementation in Zimbabwe and countries with similar settings.

## Conclusion

We found low retention at 1, 3, 6 months and delivery, a threat to PMTCT elimination agenda in Zimbabwe. Our findings emphasize the need for enhanced interventions to improve early retention such as enhanced post-test counselling, patient tracing and visit reminders.

## Data Availability

The datasets used and/or analysed during the current study are available from the corresponding author on reasonable request.

## Abbreviations

ANC: Antenatal Care
ART: Antiretroviral therapy
CI: Confidence interval
DFID: Department for International Development
EAR: Enrollment, adherence and retention
EHR: Electronic Health Records
EPMs: Electronic Monitoring System
EMTCT: Elimination of Mother-toChild Transmission (of HIV and syphilis)
GFATM: Global Fund to Fight AIDS, Tuberculosis and Malaria
HIV: Human Immunodeficiency Virus
MCH: Maternal Child Health
MoHCC: Ministry of Health and Child Care
MTCT: Mother-to-child Transmission
OI: Opportunistic Infections
PMTCT: Prevention of Mother-to-child Transmission of HIV
TB: Tuberculosis
UNAIDS: Joint United Nations Program on HIV and AIDS
WLHIV: Women Living With HIV
WHO: World Health Organisation
